# Defining the Optimal Total Number of Chemotherapy Courses in Younger Patients With Acute Myeloid Leukemia: A Comparison of Three Versus Four Courses

**DOI:** 10.1200/JCO.20.01170

**Published:** 2020-12-23

**Authors:** Alan K. Burnett, Nigel H. Russell, Robert K. Hills, Stephen Knapper, Sylvie Freeman, Brian Huntly, Richard E. Clark, Ian F. Thomas, Lars Kjeldsen, Mary Frances McMullin, Mark Drummond, Jonathan Kell, Ruth Spearing

**Affiliations:** ^1^ Department of Haematology, Cardiff University School of Medicine, Cardiff, United Kingdom; ^2^ Department of Haematology, Nottingham University Hospital NHS Trust, Nottingham, United Kingdom; ^3^ Nuffield Department of Population Health, University of Oxford, Oxford, United Kingdom; ^4^ Department of Haematology, School of Medicine, Cardiff University, Cardiff, United Kingdom; ^5^ Department of Clinical Immunology, University of Birmingham, Birmingham, United Kingdom; ^6^ Department of Haematology, and Wellcome Trust-MRC Cambridge Stem Cell Institute, University of Cambridge, Cambridge, United Kingdom; ^7^ Department of Haematology, Royal Liverpool University Hospital, Liverpool, United Kingdom; ^8^ Centre for Trials Research, Cardiff University School of Medicine, Cardiff, United Kingdom; ^9^ Department of Haematology, Rigshospitalet, Copenhagen, Denmark; ^10^ Department of Haematology, Centre for Medical Education, Queen's University, Belfast City Hospital, Belfast, United Kingdom; ^11^ Department of Haematology, Beatson Cancer Centre, Glasgow, United Kingdom; ^12^ Department of Haematology, University Hospital of Wales, Cardiff, United Kingdom; ^13^ Canterbury District Health Board, Canterbury, New Zealand

## Abstract

The optimum number of treatment courses for younger patients with acute myeloid leukemia (AML) is uncertain. The United Kingdom National Cancer Research Institute AML17 trial randomly assigned patients who were not high risk to a total of three versus four courses.

**PATIENTS AND METHODS:**

Patients received two induction courses based on daunorubicin and cytarabine (Ara-C), usually with gemtuzumab ozogamicin. Following remission, 1,017 patients were randomly assigned to a third course, MACE (amsacrine, Ara-C, and etoposide), plus a fourth course of MidAc (mitoxantrone and Ara-C) and following an amendment to one or two courses of high-dose Ara-C. Primary end points were cumulative incidence of relapse (CIR), relapse-free survival (RFS), and overall survival (OS). Outcomes were correlated with patient characteristics, mutations, cytogenetics, induction treatments, and measurable residual disease (MRD) postinduction.

**RESULTS:**

In logrank analyses, CIR and RFS at 5 years were improved in recipients of four courses (50% *v* 58%: hazard ratio [HR] 0.81 [0.69-0.97], *P* = .02 and 43% *v* 36%: HR 0.83 [0.71-0.98], *P* = .03, respectively). While OS was not significantly better (63% *v* 57%: HR 0.84 [0.69-1.03], *P* = .09), the noninferiority of three courses to four courses was not established. The impact on relapse was only significant when the fourth course was Ara-C. In exploratory analyses, although MRD impacted survival, a fourth course had no effect in either MRD-positive or MRD-negative patients. A fourth course was beneficial in patients who lacked a mutation of *FLT3* or *NPM1*, had < 3 mutations in other genes, or had a presenting WBC of < 10 × 10^9^ L^−1^.

**CONCLUSION:**

Although a fourth course of high-dose Ara-C reduced CIR and improved RFS, it did not result in a significant OS benefit. Subsets including those with favorable cytogenetics, those lacking a mutation of *FLT3 or NPM1*, or those with < 3 other mutations may derive survival benefit.

## INTRODUCTION

A number of different induction schedules and combinations given to patients with acute myeloid leukemia (AML) will achieve morphological marrow blast clearance in 75%-85% of younger patients, arbitrarily defined as < 60 years of age.^1-4^ More intensive combinations often only require a single induction course.^4^ In remission, the risk of relapse is based on several factors including presenting WBC count, age, secondary disease, morphological response of the bone marrow if not in remission, cytogenetics, and mutation analysis.^5,6^ More recently, estimates of minimal residual disease (MRD) assessed after the first or second induction course have also been shown to be important for relapse risk,^7-9^ although further data are required to establish when the MRD status is predictive of what the optimal treatment should be. From the combined prognostic information, patients will be regarded as at high, intermediate, or low risk of relapse. Usually, the issue requiring clarification is whether a stem cell transplant should be recommended. Prospective studies have now demonstrated that for patients who have *FLT3* mutation type 1 inhibitors, midostaurin^10^ but not lestaurtinib^11^ will reduce relapse risk and improve survival. Other recently approved drugs may deliver further reductions in relapse for particular subgroups.

**CONTEXT**

**Key Objective**
After two courses of induction treatment, patients who were in remission and not high risk were randomly assigned to one or two more courses of treatment.
**Knowledge Generated**
Patients given two courses of high-dose cytarabine (ie, a total of four courses) had a reduced cumulative incidence of relapse and an improved relapse-free survival but did not result in a significant benefit in overall survival.
  The trial did not establish that three courses of chemotherapy were noninferior to the standard of care of four courses.
  Although measurable residual disease (MRD) predicted the risk of relapse, overall administration of a fourth course had no survival benefit on either MRD-positive or MRD-negative patients.
**Relevance**
This study contributes information to the question of how much chemotherapy is required for younger patients with acute myeloid leukemia.

High-dose cytarabine (Ara-C) has been an established standard of care for consolidation following the landmark study of Mayer et al,^12^ who showed that a higher dose of 3 g/m^2^ was superior to 400 mg/m^2^ or 100 mg/m^2^ each for four courses after which four courses of maintenance were given.

United Kingdom trials have tried to establish the optimum number of consolidation courses since we explored giving two versus six courses in AML8.^13^ In order to explore the dose level of cytarabine and the total number of courses required, in our consecutive Medical Research Council (MRC) AML12 and 15 trials, we randomly assigned > 1,300 patients between four and five courses of total chemotherapy and established that adding a fifth course (Ara-C 1.5 g/m^2^) provided no additional benefit.^14,15^ We also demonstrated that patients randomly assigned to Ara-C 1.5 g/m^2^ for the third and fourth courses did not have a significantly inferior survival outcome to those receiving Ara-C 3 g/m^2^, either overall or in any risk subgroup.^15^ We now report the experience in the National Cancer Research Institute AML17 trial, which compared three courses versus four courses of total treatment for non–high-risk patients.

## PATIENTS AND METHODS

### The AML17 Trial

**FIG 1.**
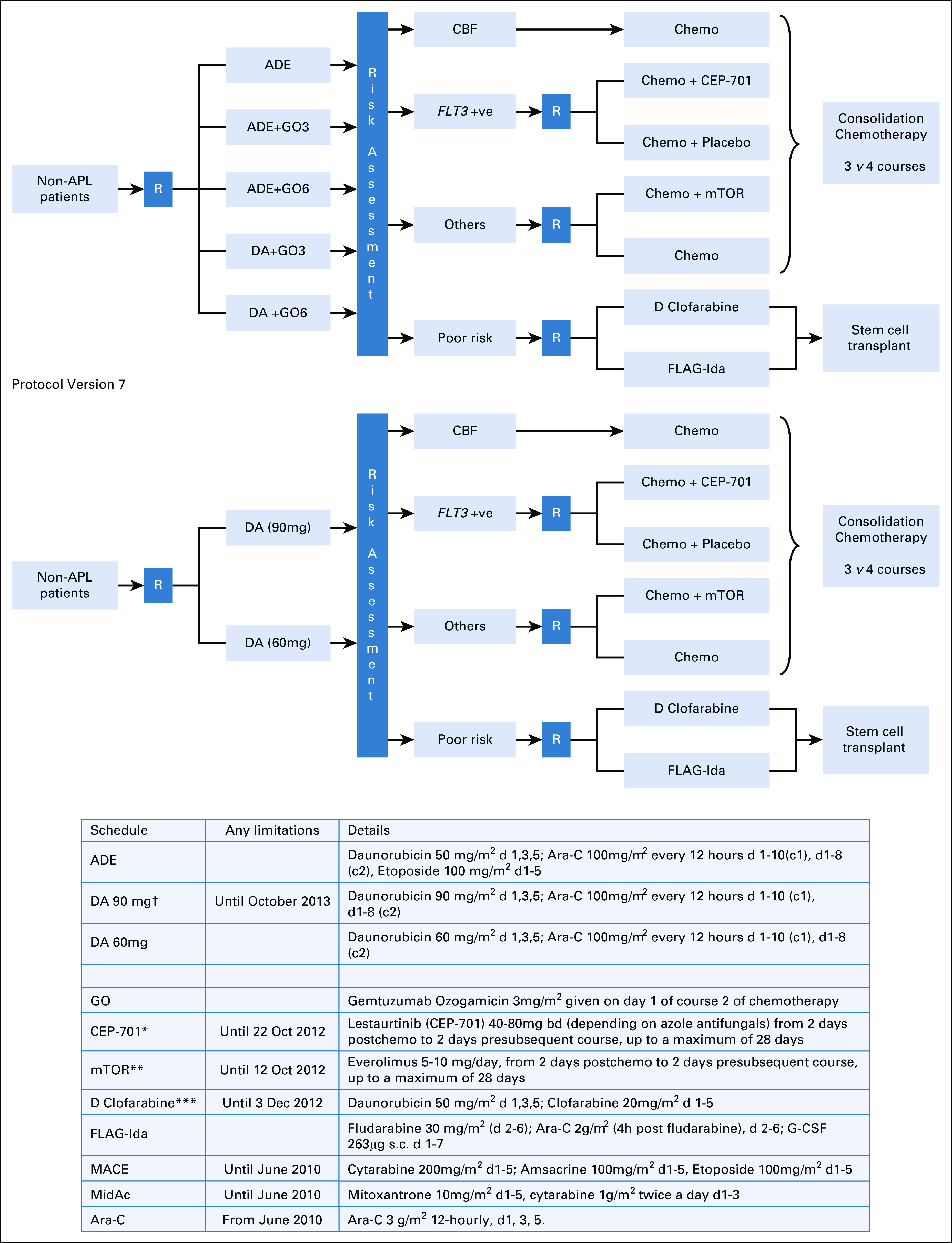
Protocol flow diagram. *Following closure of the CEP-701 randomly assigned, patients were guided by risk score to either poor risk or nonpoor risk options. **Following closure of the mTOR inhibition random assignment, patients in this group received DA 50mg alone. ***Following closure of the D Clofarabine arm, patients were recommended to receive FLAG-Ida (which was also the case if renal criteria were not met). †Following closure of the high-dose daunorubicin arm, patients were allocated DA60. ADE, Ara-C, daunorubicin, and etoposide; APL, acute promyelocytic leukemia; CBF, core binding factor; DA, daunorubicin and Ara-C; FLAG-Ida, fludarabine, Ara-C, granulocyte colony-stimulating factor, and idarubicin; GO, gemtuzumab ozogamicin; MACE, amsacrine, Ara-C, and etoposide; MidAc, mitoxantrone and Ara-C; mTor, mammalian target of Rapamicin.

The United Kingdom MRC AML17 trial (ISRCTN55675535) tested a number of interventions for induction, which have already been reported.^11,16-18^ Briefly, patients of age from 18 years usually up to 60 years were initially randomly assigned to receive ADE (Ara-C, daunorubicin, and etoposide) or DA (daunorubicin and Ara-C) for the first two courses combined with gemtuzumab ozogamicin as a single dose of 3 mg/m^2^ or 6 mg/m^2^ in course 1, in which neither the addition of etoposide nor dosing of gemtuzumab ozogamicin at 6 mg/m^2^ improved the result.^18^ In a subsequent amendment, induction treatment was DA treatment where the daunorubicin dose in course 1 was either 60 mg/m^2^ or 90 mg/m^2^. In this comparison, no overall difference was found,^4^ but it later emerged that patients with a *FLT3* mutation benefited from the dose of 90 mg/m^2^.^19^ Patients with high-risk myelodysplastic syndrome (defined as > 10% marrow blasts), with de novo or secondary AML, with any WHO performance score could be included, but the blast transformation of chronic myeloid leukemia and acute promyelocytic leukemia was excluded. After the first course of induction treatment, patients were designated as high-, intermediate-, or low-risk based on our validated weighted risk score,^5,6^ which is based on the presenting WBC count, age, cytogenetics, and secondary disease and is presented in detail in the Protocol (online only). High-risk patients were subjected to a separate random assignment of FLAG-Ida (fludarabine, Ara-C, granulocyte colony-stimulating factor, and idarubicin) versus DClo (daunorubicin and clofarabine) with the intention to proceed to transplantation, which has previously been reported,^16^ showing the superiority of FLAG-Ida in this setting. Intermediate-risk patients with a *FLT3* mutation could enter a random assignment of the addition of the *FLT3* inhibitor, lestaurtinib, or not, while other intermediate-risk patients without the *FLT3* mutation could be randomly assigned to the addition of the mammalian target of Rapamicin inhibitor, everolimus, or not. The results of both interventions have previously been reported with neither addition showing overall benefit.^11,17^ After the two induction courses, all intermediate- and good-risk patients, whether receiving lestaurtinib or everolimus or not, were eligible to be randomly assigned to have one or two consolidation courses following the confirmation of remission (ie, three *v* four courses of treatment in total). This random assignment helped in recruiting patients from April 2009 to December 2014. Initially, until June 2010, the consolidation treatment random assignment for the third and fourth courses was between MACE (amsacrine, Ara-C, and etoposide) and MACE plus MidAc (mitoxantrone and Ara-C) (n = 120). In light of the results of the previous MRC AML15 trial, which compared MACE/MidAc with two courses of Ara-C,^15^ a subsequent protocol amendment changed the random assignment to one versus two courses of high-dose Ara-C (3 g/m^2^ twice a day days 1, 3, and 5) (n = 897). Random assignment took place after count recovery following the second induction course. The aim of the three versus four random assignment was to define if a fourth treatment course was necessary and whether the treatments involved were relevant. The trial flow diagram and details of drugs used are shown in Fig 1.

### Correlative Studies

Cytogenetic analyses were undertaken locally in laboratories that participate in the national quality assurance scheme, centrally reviewed, and classified according to our established criteria.^20^ Mutation analysis of the *FLT3 and NPM1* status was performed in a single reference lab. Although not integral to therapeutic decisions in the trial, samples for MRD, which were not disclosed to investigators, were collected after each induction course and undertaken by flow cytometry in one of the two reference labs by methods previously described,^8,21^ and whole genome sequencing (Sanger sequencing) as described elsewhere^22^ of additional 82 genes was undertaken on 443 stored samples from participants in this random assignment at the Sanger Centre (Cambridge, United Kingdom). The *FLT3* mutation status was provided during the trial to enable entry to the lestaurtinib random assignment.

Patients were randomly assigned in 110 centers in the United Kingdom, five in Denmark, and five in New Zealand. The trial was sponsored by Cardiff University and approved by the All Wales Research Ethics Committee on behalf of all UK investigators, by the Danish Medicines Agency for sites in Denmark, and by the New Zealand Medicines and Medical Devices Safety Agency for sites in New Zealand.

Written consent was obtained for each random assignment and for the storage of diagnostic samples. The trial was conducted in accordance with the Declaration of Helsinki.

### Statistical Considerations and End Points

The primary outcome measure for this random assignment was overall survival (OS) at 5 years. It was anticipated that about 55% of patients who entered the whole AML17 trial would be available for the consolidation chemotherapy random assignment. The trial was anticipated to detect, with 90% power, a difference in survival from 55% to 65%, equivalent to a hazard ratio (HR) of 0.71; a critical number of 370 events was required to evaluate this difference. The primary question was whether three courses were noninferior to four courses, at a one-sided significance of 0.025; consequently, effect sizes are reported with 95% two-sided CIs throughout. Noninferiority would be concluded if the lower 95% CI bound was above 0.71. Toxicity (hematologic recovery times and nonhematologic toxicity) was scored using the National Cancer Institute Common Toxicity Criteria, Version 3, and resource use data (blood product support, days on antibiotics, and hospitalization) were collected. All end points were defined according to the revised International Working Group criteria,^23^ where OS and relapse-free survival (RFS) were measured from the point of random assignment.

The analyses are by intention to treat. Categorical end points (eg, OS) were compared using Mantel-Haenszel tests, giving Peto odds ratios (ORs) and CIs. Continuous/scale variables were analyzed by nonparametric (Wilcoxon rank-sum) tests. Time-to-event outcomes were analyzed using the logrank test, with Kaplan-Meier survival curves. Analyses adjusted for random assignment parameters are performed using the Cox regression and given in parallel to the assumption-free logrank approach. ORs/HRs < 1 indicate benefit for the extra course of chemotherapy. All survival percentages are at 5 years unless otherwise stated. The median follow-up at the time of final analysis was 55.1 months (range, 1.2-99.4 months).

In addition to overall analyses, exploratory analyses were performed stratified by the random assignment stratification parameters and other important variables, including correlations with MRD, with suitable tests for interaction. Because of the well-known dangers of subgroup analysis, these were interpreted cautiously.

## RESULTS

### Patient Characteristics

**FIG 2.**
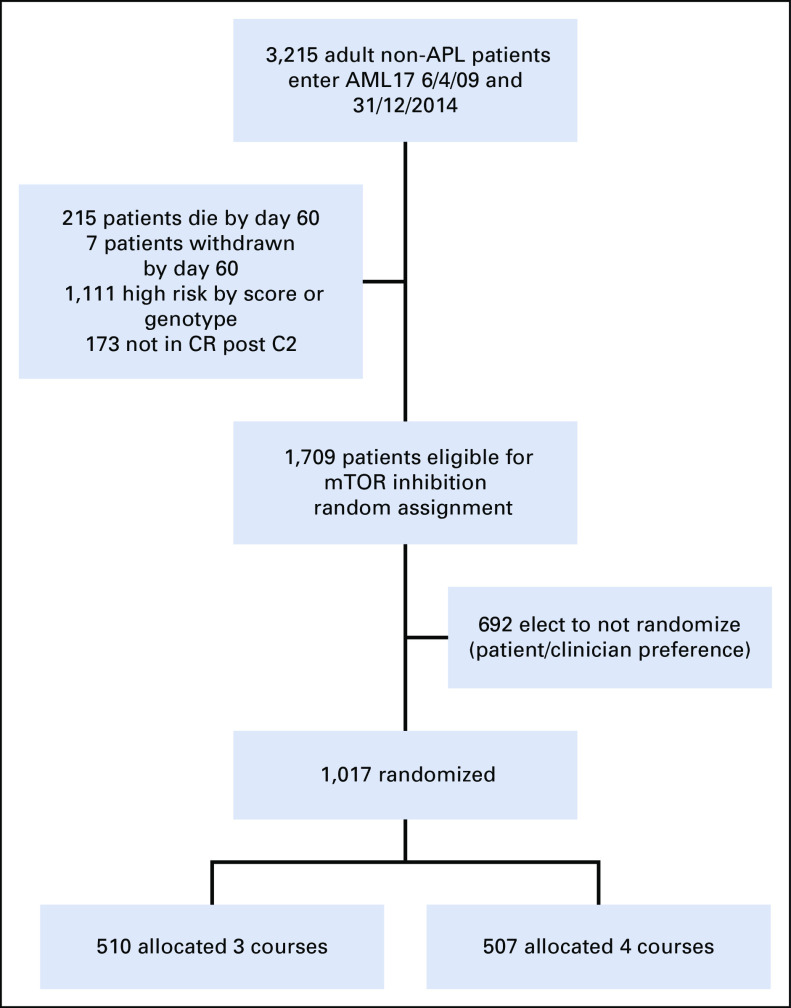
CONSORT diagram. AML, acute myeloid leukemia; APL, acute promyelocytic leukemia; CR, complete remission; mTor, mammalian target of Rapamicin.

Between April 2009 and December 2014, a total of 1,709 patients, on recovery from induction course 2, were eligible for this random assignment of whom 1,017 (60%) were randomly assigned. The reasons for not being randomly assigned were only listed as patient or clinician preference. The interval between diagnosis and random assignment was 2.6 months (range, 1.4-5.2 months). Patients not entering the random assignment were generally similar but were less likely to be de novo AML, to have worse cytogenetics, and to have received DA60 in induction (Protocol); however, the OS at 5 years of those eligible who reached the median time of random assignment but did not enter the random assignment was 60%, which was the same for those who were randomly assigned (60%; *P* = .4). The characteristics and treatments of the randomly assigned patients are shown in Table 1. There were no differences between those randomly assigned with respect to age, sex, performance score, presenting WBC count, cytogenetic risk group, *NPM1* status, induction treatments, and risk score or number given stem cell transplant overall or in CR1. There was a modest difference in 479 patients whose MRD status was known after course 1 or in 365 randomly assigned patients whose MRD status was known after course 2, with fewer patients allocated to three courses to be MRD-negative. As previously stated, the MRD status of patients was not made available to investigators. There was no difference in the frequency of mutations or in the numbers of patients with different numbers of mutations detected. The deployment of patients is shown in Fig 2.

**TABLE 1.**
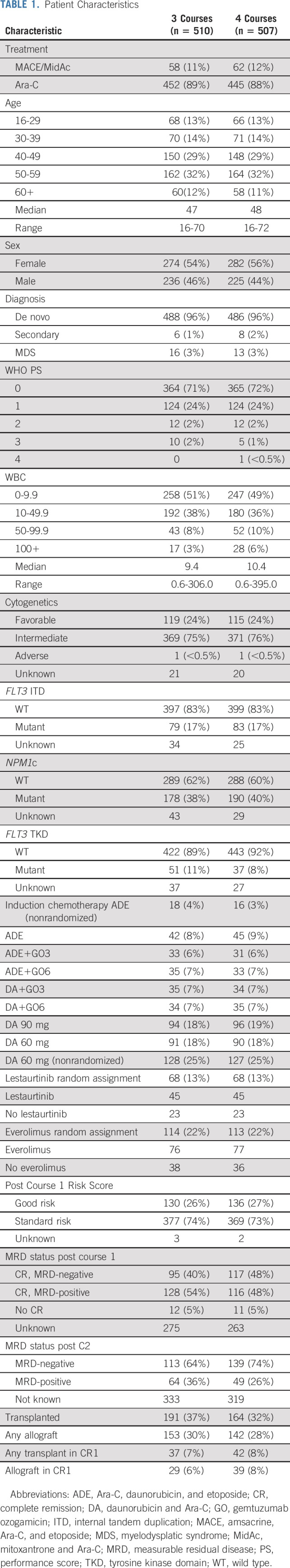
Patient Characteristics

Although the addition of a fourth course significantly reduced the cumulative incidence of relapse (CIR) and RFS, the OS difference at 5 years failed to reach significance.

### CIR and RFS

**FIG 3.**
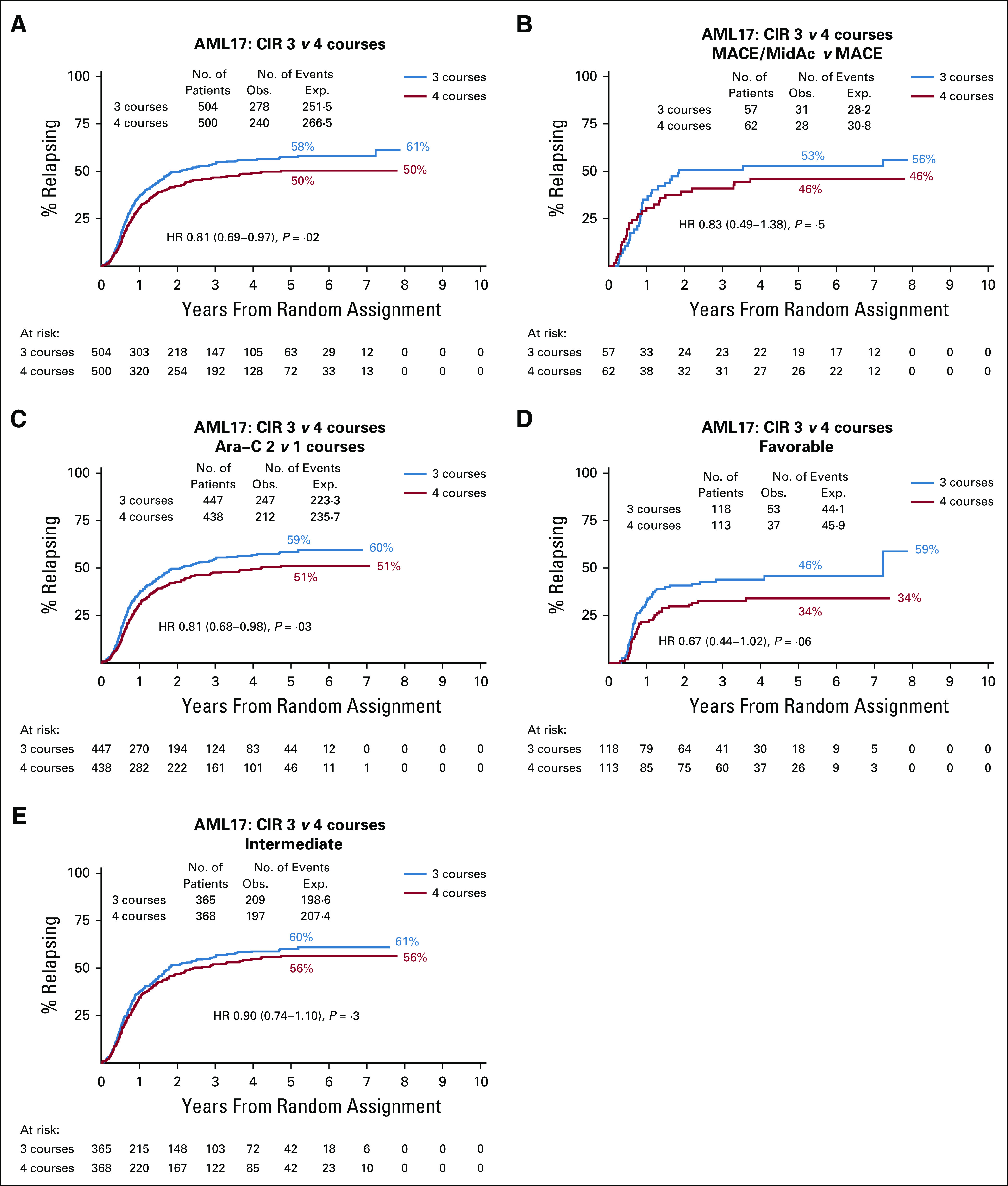
Cumulative incidence of relapse. AML, acute myeloid leukemia; CIR, cumulative incidence of relapse; HR, hazard ratio; MACE, amsacrine, Ara-C, and etoposide; MidAc, mitoxantrone and Ara-C.

Overall, those allocated to the fourth course had a significantly lower CIR: 50% *v* 58% (raw HR 0.81 [0.69-0.97], *P* = .02; adjusted HR 0.82 [0.69-0.97], *P* = .02) (Data Supplement and Fig 3A). With respect to the treatment received, there was no significant interaction. While the effect on CIR was only significant with Ara-C as the fourth course (Figs 3B-3C), effect sizes are similar (0.82 [0.49-1.38] MACE/MidAc, *v* 0.81 [0.68-0.98] Ara-C). Similarly, while the effect appeared stronger in favorable-risk patients (0.67 [0.45-1.02]) than those with intermediate cytogenetics (0.90 [0.74-1.10]), there was no significant interaction (*P* = .2, Figs 3D-3E).

Overall RFS differences, by treatment and risk groups, showed the same differences as CIR (Protocol), namely that there was benefit in a fourth course with respect to prevention of relapse.

### Treatment Compliance

Of the 507 patients allocated to four courses, 74% received all intended courses, which resulted in a 67% survival, whereas for those allocated four but who only received three, survival was 54% (*P* = .002; HR, 0.58 [0.040-0.84]). The recipients of four courses required a median time of an extra 23 days of hospitalization, 9 days on antibiotics, 5.6 and 5.9 units of RBCs and platelets and 4 and 5 weeks for recovery of neutrophils and platelets, and had a 2% risk of death within 60 days.

### OS

**FIG 4.**
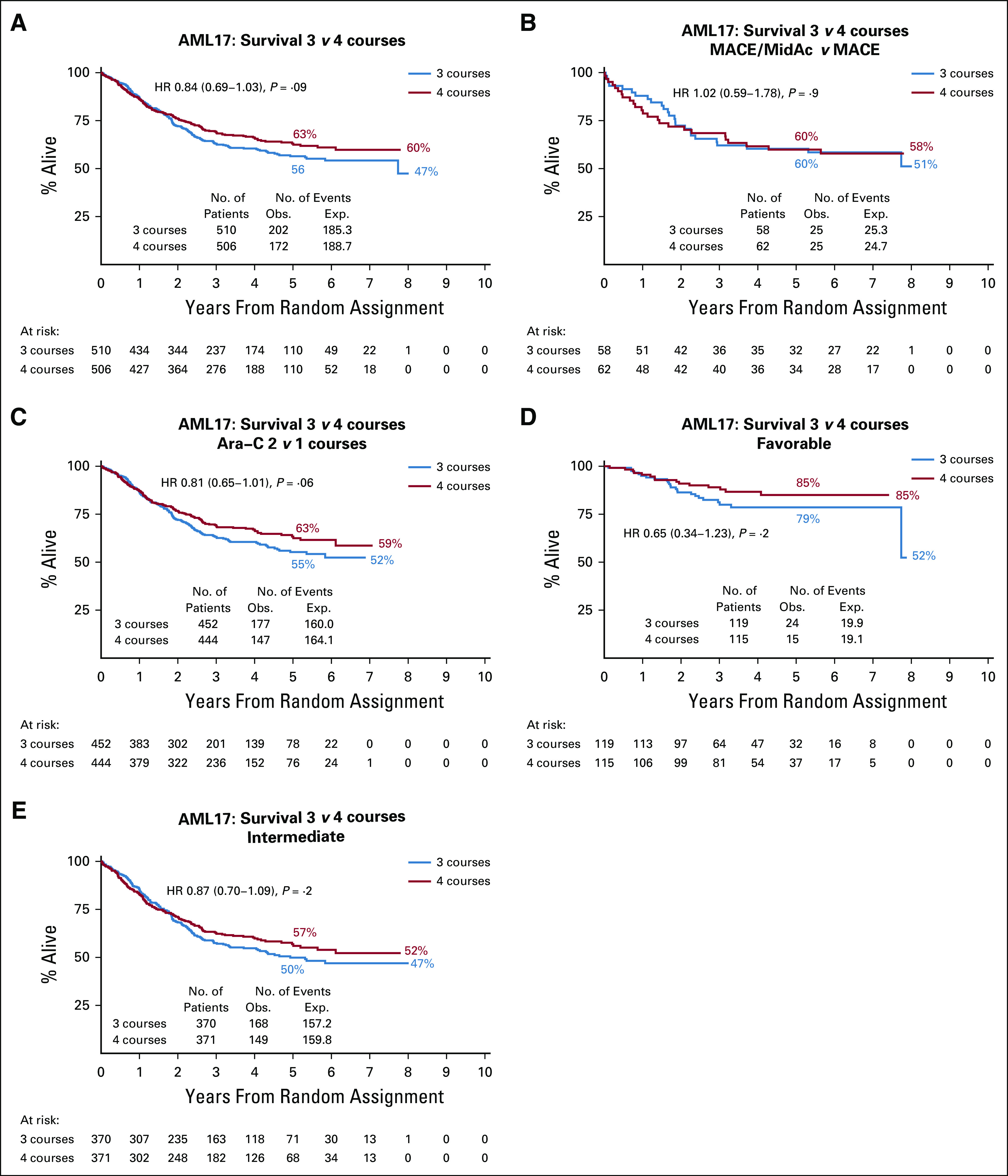
Overall survival. AML, acute myeloid leukemia; HR, hazard ratio; MACE, amsacrine, Ara-C, and etoposide; MidAc, mitoxantrone and Ara-C.

There was a nonsignificant difference in favor of four courses (63% *v* 56%) with respect to survival (Fig 4A). Considering the question of whether three courses were noninferior to four courses, the CI in both unadjusted and adjusted analyses crosses the threshold of 0.71, meaning that there is no evidence to conclude noninferiority at this threshold. In spite of the reduced CIR, there was no detectable survival difference in patients treated in the MACE/MidAc arm (Fig 4B), but there were nonsignificant differences in favor of four courses in patients treated in the Ara-C arms in both risk groups (Figs 4C-4E). If the 79 patients who received a transplant in CR1 are censored at transplant, the survival rates are 63% for three courses and 72% for four courses. The overall outcomes are summarized in the Protocol.

### Exploratory Subgroup Analyses

**FIG 5.**
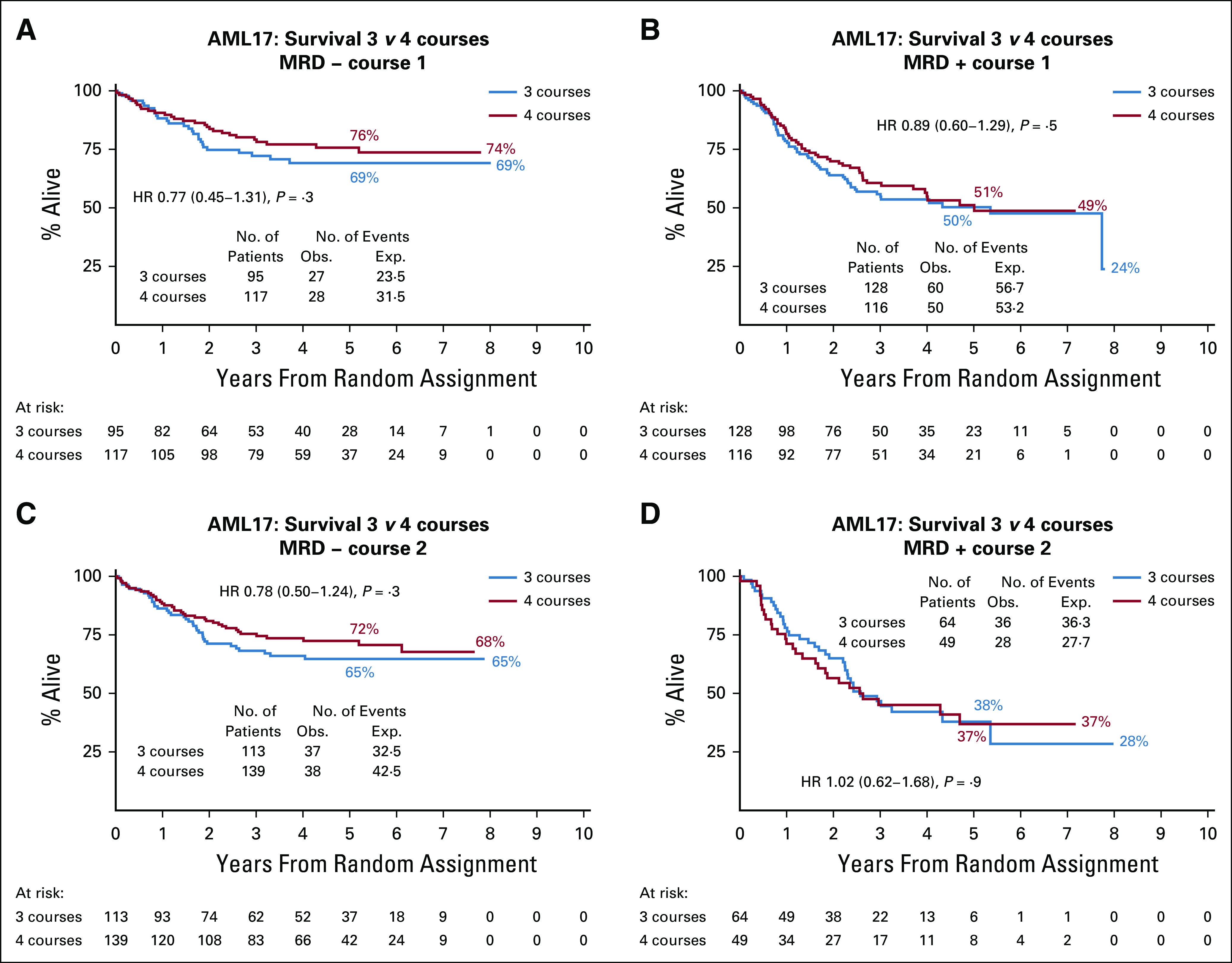
Effect of measurable residual disease (MRD). AML, acute myeloid leukemia; HR, hazard ratio.

The outcome was not affected by any of the patients' characteristics or induction treatments (Protocol), although those presenting with a low WBC of < 10.0 × 10^9^/L had a significant benefit from four courses. MRD information was obtained after course 1 in 456 patients and in 365 patients after course 2. The OS at 5 years in patients who were MRD-negative after the first or second induction courses (n = 464) at 73% was better than that in the MRD-positive patients (n = 357) at 50%. In patients who were assessed after course 1 of induction, the OS was not significantly different between the treatment arms, irrespective of the MRD status (Figs 5A-5B). In patients with MRD information after course 2, the OS was 69% if MRD-negative and 37% if MRD-positive, but again there was no significant difference in either groups if allocated to three or four courses (Figs 5C-5D).

Although there appears to be a significant advantage of four courses in patients who received daunorubicin 90 mg/m^2^ in induction (Protocol), the test for heterogeneity was not significant, suggesting that any apparent benefit is not conclusive.

**FIG 6.**
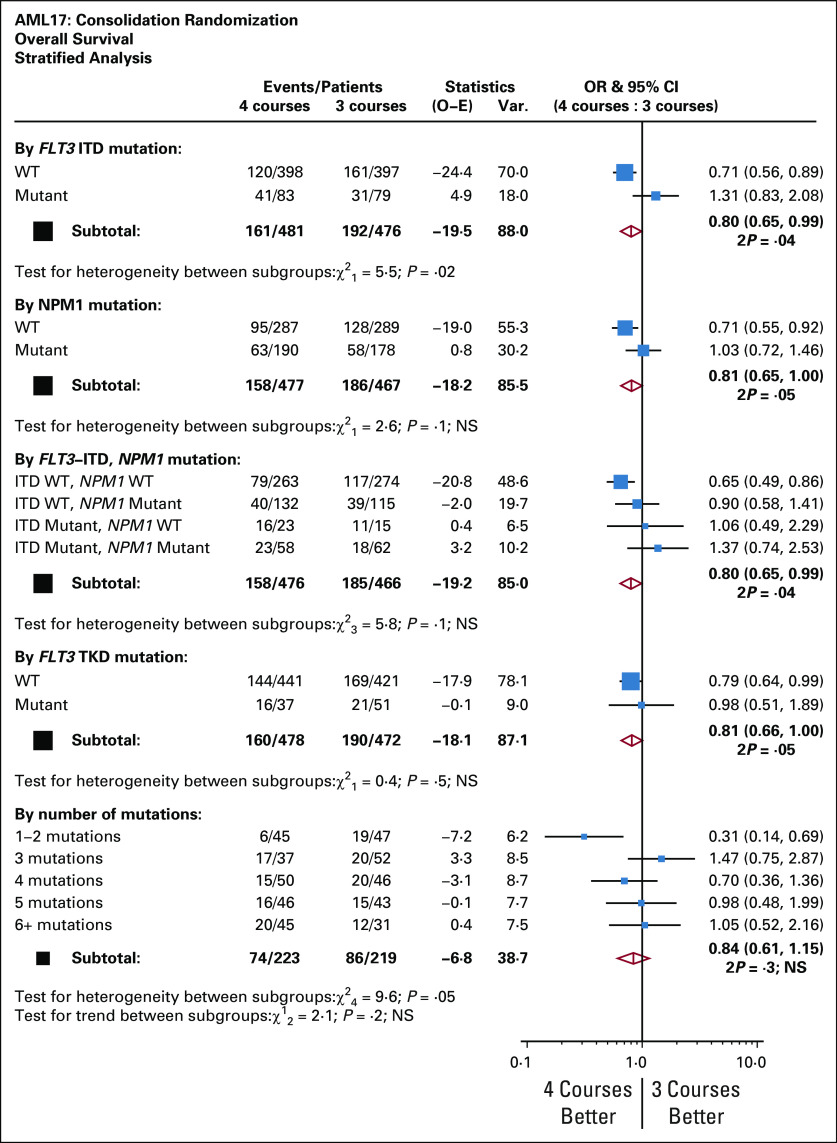
Analysis stratified by mutation status. AML, acute myeloid leukemia; ITD, internal tandem duplication; OR, odds ratio; TKD, tyrosine kinase domain; WT, wild type.

Four courses were significantly beneficial in patients without an *FLT3* internal tandem duplication or tyrosine kinase domain or *NPM1* mutation or in 92 of the 433 patients with < 3 mutations as detected using Sanger sequencing (Fig 6). The benefit appeared greatest in patients with *FLT3/NMP1* wild type, although there was no significant interaction. For the purpose of assessing the prognostic value of the mutations detected using Sanger sequencing, only mutations that occurred in more than 20 of the 433 patients were considered, but no correlations were observed (Protocol).

## DISCUSSION

The recent approvals of new drugs for AML may move the treatment algorithm in the relevant subgroups. However, it is yet to be established for some of these new drugs whether combination with standard chemotherapy may be their optimal use.^24^ It therefore remains important to define the optimal total treatment with chemotherapy that is required. There has been extensive effort to establish the best agent and doses for induction, but less attention to the dose and number of courses of postinduction treatment. Recruiting sufficiently large numbers to reliably answer questions at this stage of treatment is a logistical challenge. Definitive studies 25 years ago established high-dose Ara-C as the standard of care for up to four courses at a dose level of 3 g/m^2^. The MRC AML15 trial (ISRCTN17161961) established that our previous standard of care (MACE plus MidAc) was superior to high-dose Ara-C in adverse-risk patients.^15^ There was little survival difference between 3 g/m^2^ and 1.5 g/m^2^ Ara-C dose levels. The addition of a fifth course was tested in the MRC AML12 and 15 trials^14,15^ with no evidence of benefit for a fifth course. A number of collaborative group trials have assessed the number of courses without providing a universally accepted conclusion.^25-27^

In this trial, random assignment took place after the completion of two induction courses and 1,709 (53%) of the original trial entrants were eligible. In the intent-to-treat analysis of the 1,017 patients randomly assigned, it emerged that the addition of the fourth course significantly reduced the CIR and significantly improved the RFS, but the trend for better OS was not significant. This was the case in the 885 patients who received exclusively Ara-C. Because of carry over random assignment from our AML15 trial, 120 patients received MACE or MACE plus MidAc among whom there was a similar trend for reduction in CIR and improvement in RFS, but neither of which reached statistical significance. Although the numbers become too small for confidence, the benefit (CIR and RFS) of the fourth course is more obvious in the favorable-risk group. In terms of OS, based on the number of patients assessable, there is a strong trend for benefit in the recipients of Ara-C, but not for the recipients of MACE/MidAc.

Among the several subgroups examined where a significant difference was observed in conjunction with a test for heterogeneity, which reached significance, were patients who lacked a *FLT3* or *NPM1* mutation, patients with one or two mutations compared with > 2, and patients with presenting WBCs of < 10 × 10^9^/L. Patients who were MRD-negative (at a level of 1 in 10^4^) after course 1 or 2 had a significantly better survival than those who were positive at either time point, but the addition of a fourth course was not beneficial in either group. In general, patients with more favorable characteristics appear to benefit from a fourth course, but only when high dose Ara-C consolidation is used, whereas those with intermediate-risk characteristics do not, although these were only trends for benefit.

There was a price to pay for the fourth course with respect to days in hospital, days on antibiotics, and blood product support although there were no excessive deaths in remission. However, as a consequence of the greater number of relapses, more salvage transplants were required in patients receiving just three courses (Table 1). It could be speculated that the most useful interaction will eventually be the initial discrimination based on the MRD status after the first or second induction course, where there may be little benefit in a fourth course for those who are MRD-positive, but benefit for those who are negative, or vice versa.

Overall, this experience suggests that if Ara-C is the chosen consolidation treatment, a fourth course of overall treatment is probably beneficial. In a retrospective analysis of the induction in the AML15 trial,^15^ patients who received two courses of FLAG-Ida as induction without consolidation had a similar survival to control patients who received two induction courses of DA treatment followed by two courses of consolidation. These were nonrandomized data and are currently being tested in the ongoing AML19 trial (ISRCTN78449203).

## Figures and Tables

**FIG A1.**
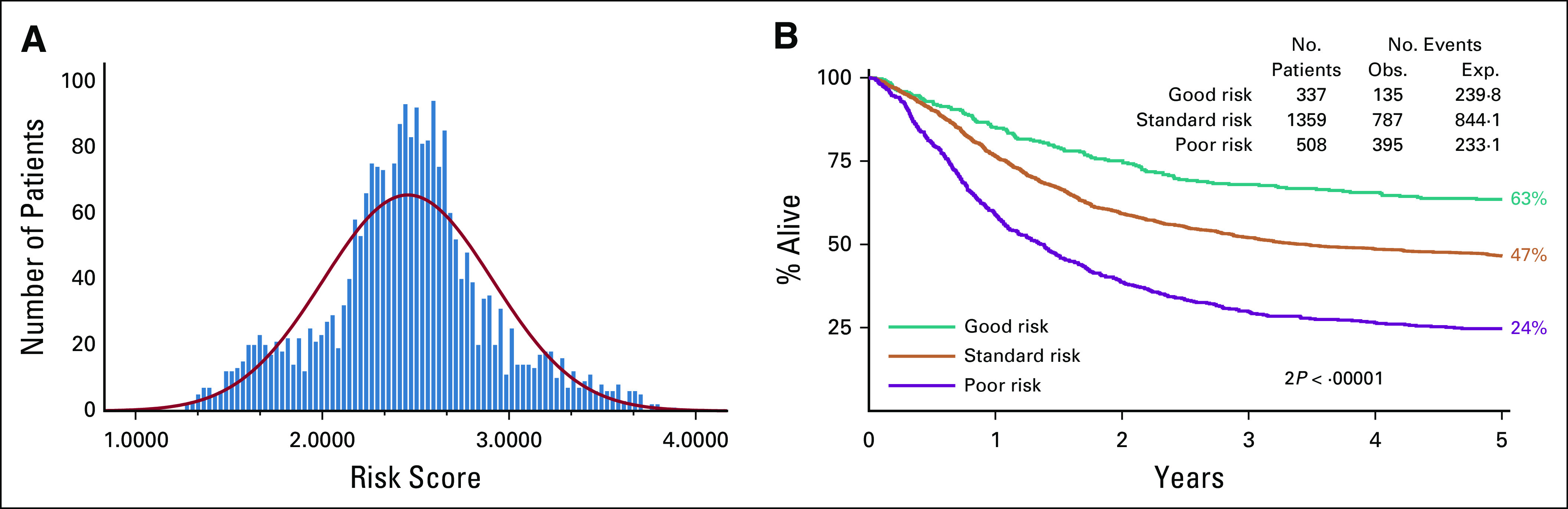
Patients' score derived from multiplying each relevant characteristic by the value derived from the Cox model: 0.01325*age (in years) + 0.16994*sex (1 = male, 0 = female) + 0.22131*diagnosis (1 = de novo, 2 secondary) + 0.65082*cytogenetics (1 = favorable, 2 = intermediate, 3 adverse) + 0.19529*status post C1 (1 = complete remission, 2 = partial remission, 3 = no response) + 0.00169* WBC (x109/l). Distribution of patients in MRC AML10,12 trials by index: Taking into account the apparent bimodality of the curve, patients with an index of 2 or below were deemed good risk, and the data were arbitrarily divided at the 75th centile between standard and poor risk. Survival index from complete remission in AML10,12 according to the risk groups was validated on data from MRC AML15 Trial.^5^

**FIG A2.**
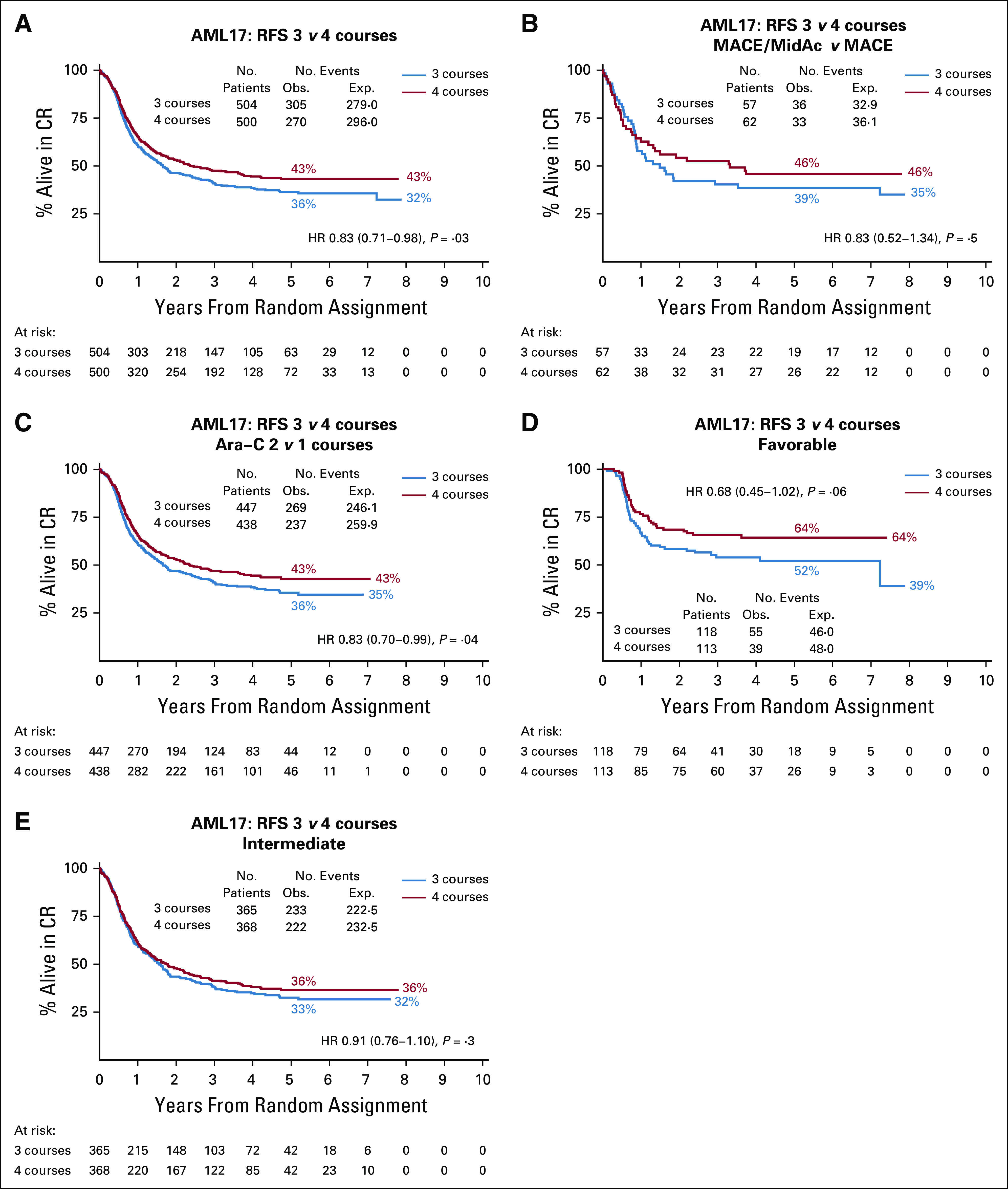
Relapse-free survival (RFS). AML, acute myeloid leukemia; CR, complete remission; MACE, amsacrine, Ara-C, and etoposide; MidAc, mitoxantrone and Ara-C.

**FIG A3.**
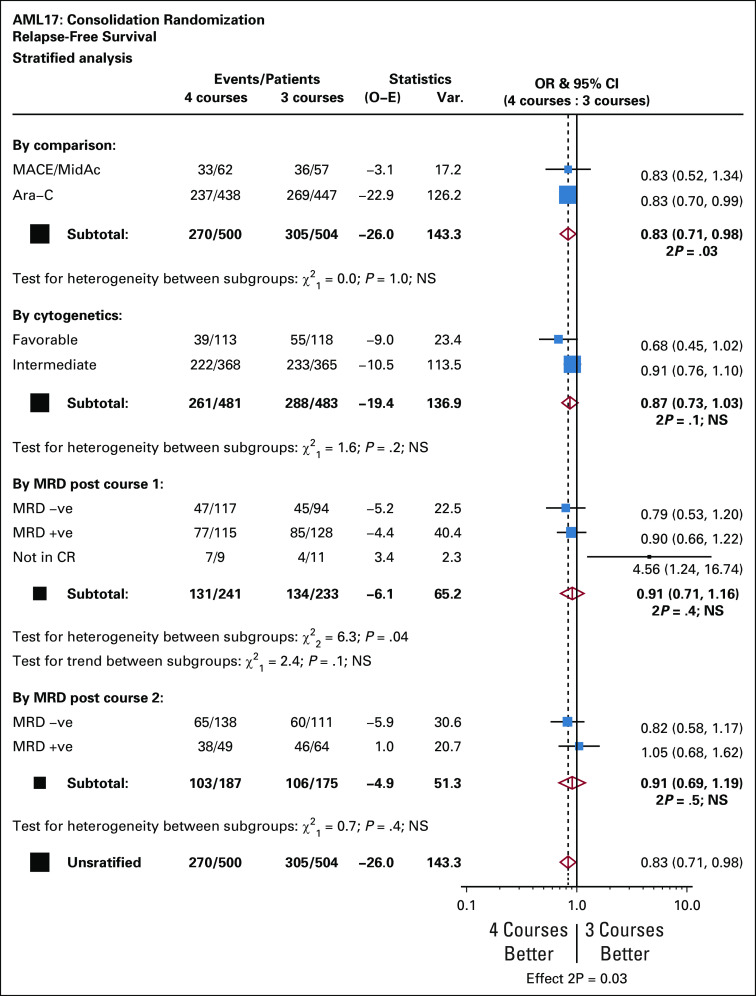
Stratified analysis of relapse-free survival. AML, acute myeloid leukemia; CR, complete remission; MACE, amsacrine, Ara-C, and etoposide; MidAc, mitoxantrone and Ara-C; MRD, measurable residual disease; OR, odds ratio.

**FIG A4.**
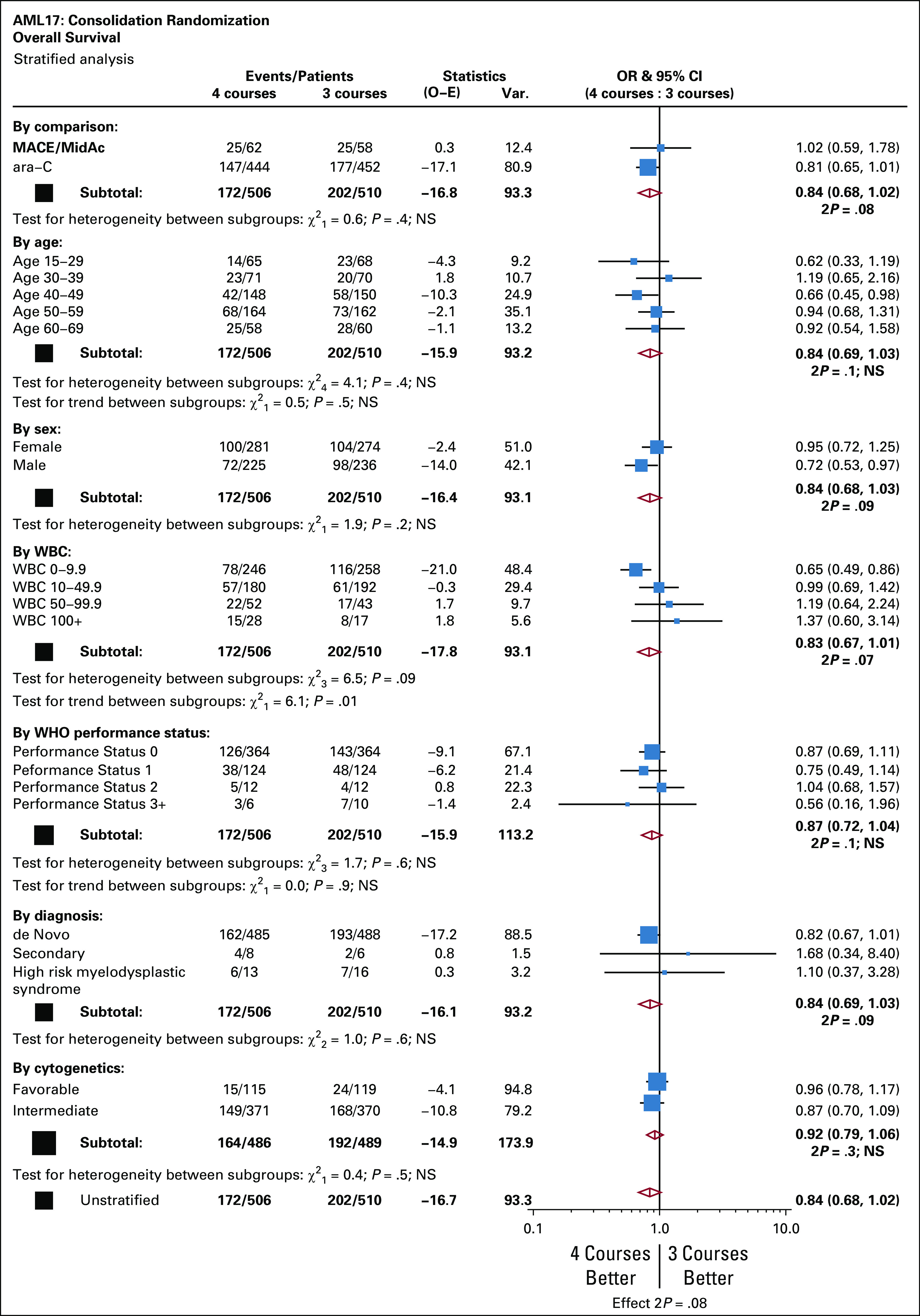
Stratified analysis of overall survival. AML, acute myeloid leukemia; CR, complete remission; MACE, amsacrine, Ara-C, and etoposide; MidAC, mitoxantrone and Ara-C; OR, odds ratio.

**FIG A5.**
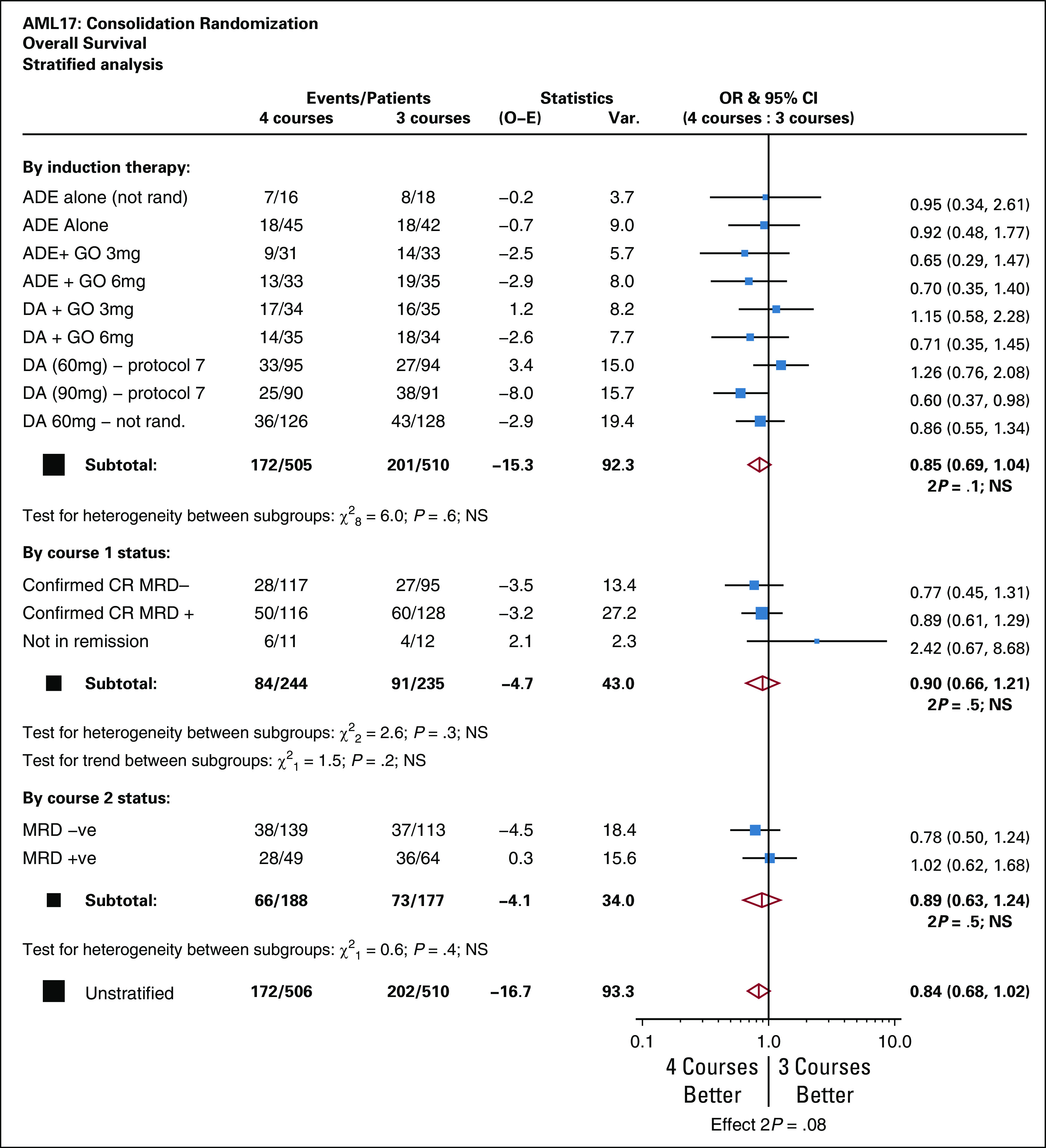
Analysis stratified by prior treatment and MRD status. ADE, Ara-C, daunorubicin, and etoposide; AML, acute myeloid leukemia; CR, complete remission; DA, daunorubicin and Ara-C; GO, gemtuzumab ozogamicin; MACE, amsacrine, Ara-C, and etoposide; MidAC, mitoxantrone and Ara-C; MRD, measurable residual disease; OR, odds ratio.

**FIG A6.**
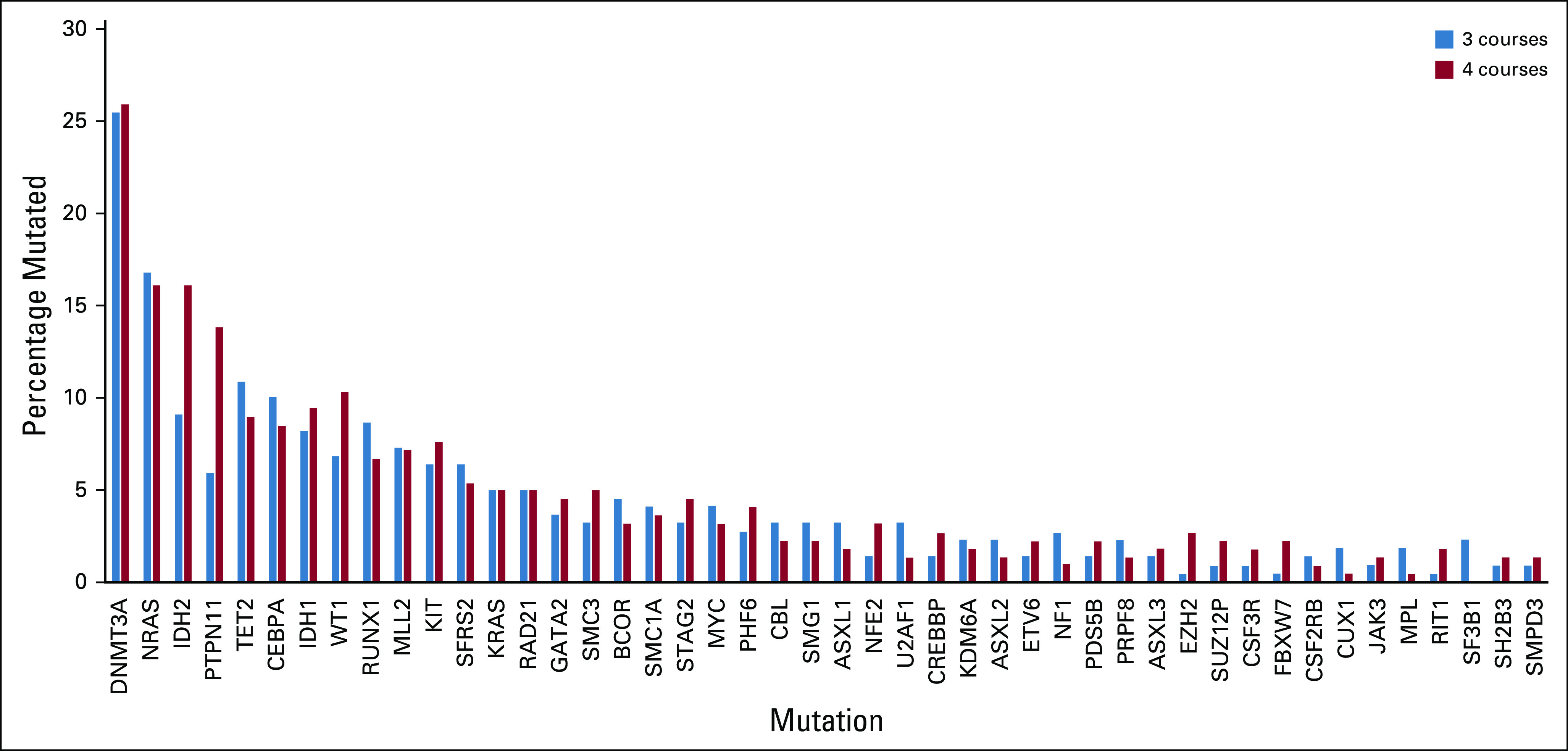
Sanger sequencing.

**FIG A7.**
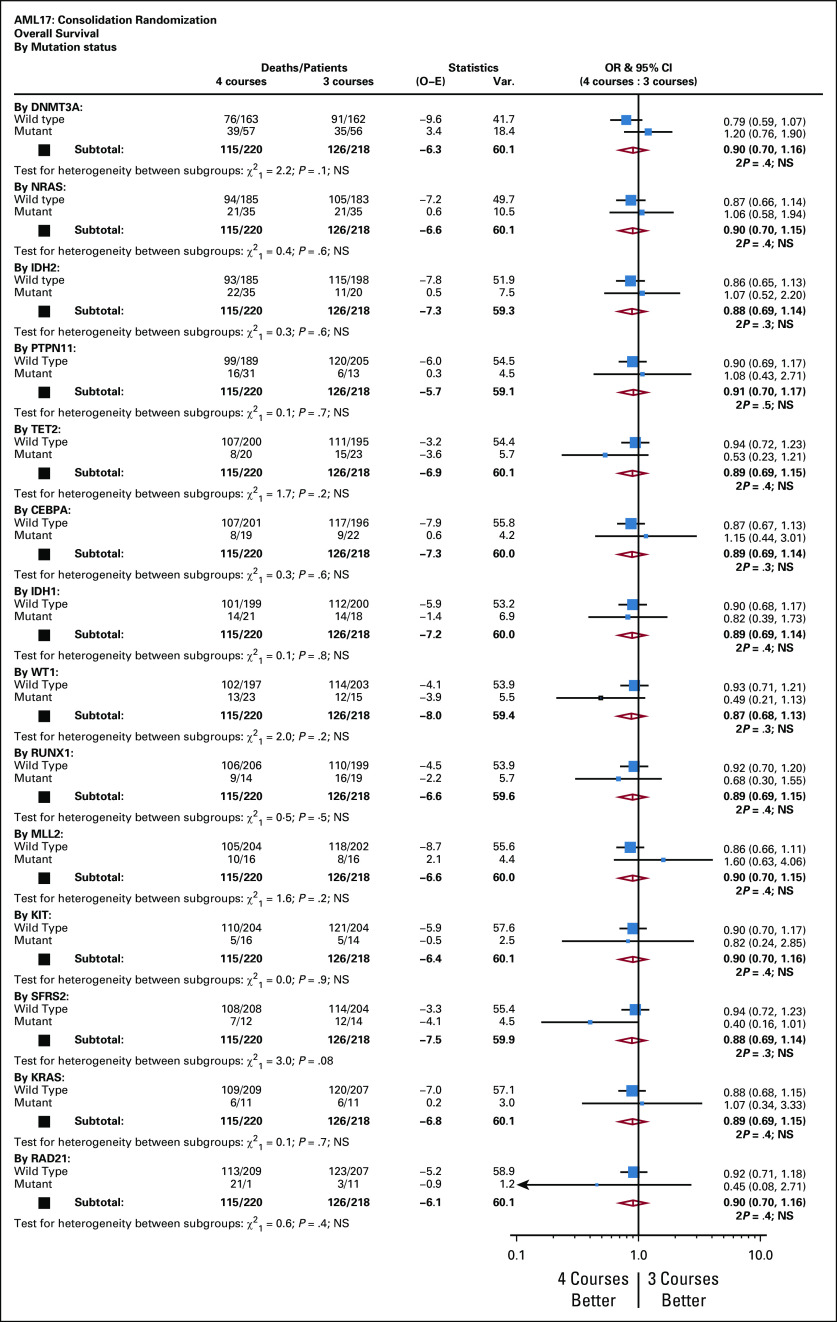
Analysis stratified by Sanger sequencing. AML, acute myeloid leukemia; OR, odds ratio.

**TABLE A1.**
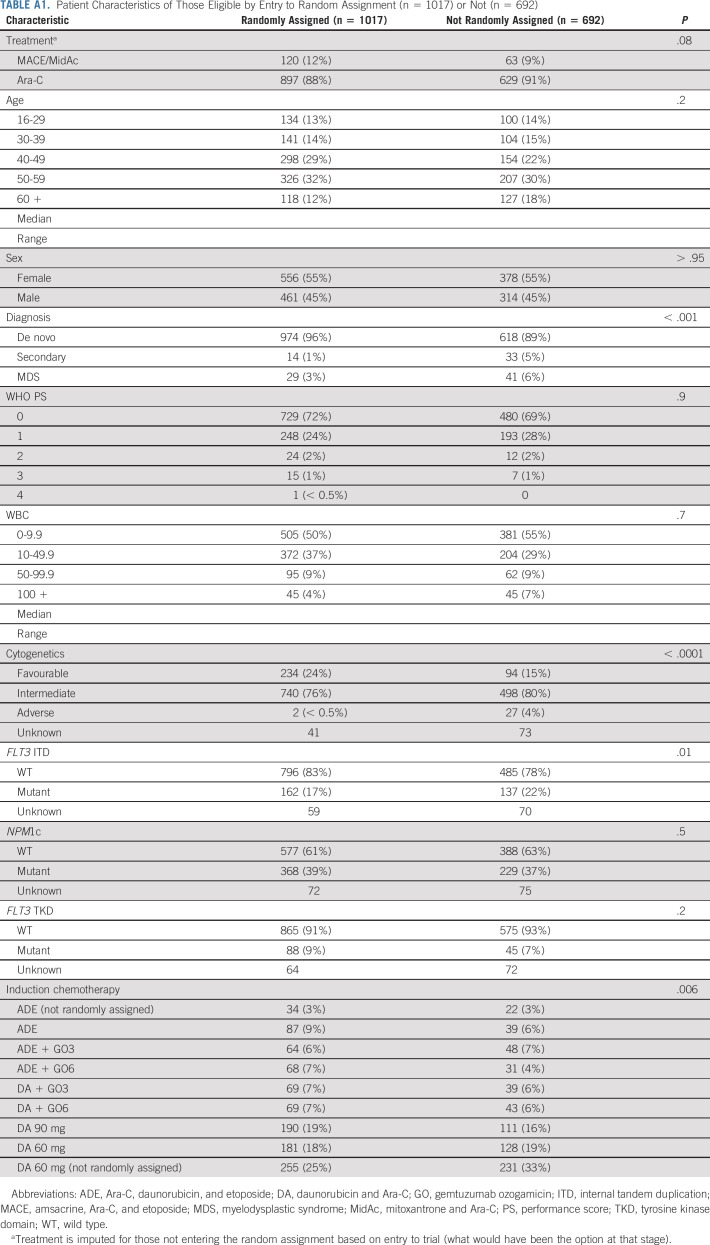
Patient Characteristics of Those Eligible by Entry to Random Assignment (n = 1017) or Not (n = 692)

**TABLE A2.**
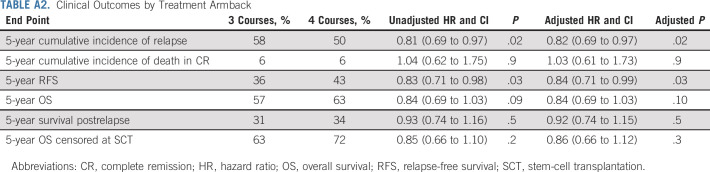
Clinical Outcomes by Treatment Armback

## APPENDIX

The following investigators recruited patients: Aalborg Hospital: Maria Kallenbach; Aarhus University Hospital: Hans Beier Ommen, Ingolf Molle, Jan Maxwell Norgaard, Mette Holm; Arrowe Park Hospital: Ranjit Dasgupta; Auckland City Hospital: Leanne Berkhan, Lucy Pemberton, Nigel Patton, Richard Doocey, Sophie Lee, Timothy Hawkins; Ayr Hospital: Fiona Cutler, Paul Eynaud; Barnet General Hospital: Andres Virchis, Sylvia Berney; Barts and the London NHS Trust: Jamie Cavenagh, Matthew Smith, Simon Hallam, Heather Oakervee; Basingstoke and North Hampshire Foundation NHS Trust: Ashok Roy, Sylwia Simpson; Beatson West of Scotland Cancer Center: Anne Parker, Mark Drummond, Mike Leach, Pam McKay, Richard Soutar; Belfast City Hospital: Claire Arnold, Damian Finnegan, Mary Frances McMullin, Robert Cuthbert, Victoria Pechey; Birmingham Heartlands Hospital: Donald Milligan, Guy Pratt, Manos Nikolousis, Matthew Lumley, Neil Smith, Richard Lovell, Shankara Paneesha; Blackpool Victoria Hospital NHS Foundation Trust: Paul Cahalin; Borders General Hospital: John Tucker; Bradford Royal infirmary: Adrian Wiliams, Anita Hill, Lisa Newton, Nitin Sood, Sam Ackroyd; Bristol Haematology and Oncology Center: David Marks, Graham Standen, Jenny Bird, Priyanka Mehta, Roger Evely; Cambridge University Hospitals NHS Foundation Trust: Charles Crawley, George Follows, Jenny Craig, Pramila Krishnamurthy; Cheltenham General Hospital: Adam Rye, Richard Lush; Chesterfield Royal Hospital: Andrew Fletcher, Mark Wodzinski, Peter Toth, Robert Cutting; Christchurch Hospital: Andrew Butler, Mark Smith, Peter Ganly, Ruth Spearing, Steve Gibbons; Christie Hospital NHS Trust: Mike Dennis; Countess of Chester hospital: Salah Tuegar; Crosshouse Hospital: Julie Gillies, Mark McColl, Peter Maclean; Darent Valley Hospital: Anil Kamat, Raphael Ezekwesili; Derby Hospitals NHS Foundation Trust: Christopher Millar, David Allotey, Juanah Addada; Derriford Hospital: Hannah Hunter, Patrick Medd, Simon Rule, Tim Nokes, Wayne Thomas; Doncaster Royal Infirmary: Joe Joseph, Robert Cutting, Stuti Kaul, Youssef Sorour; Dorset County Hospital NHS Foundation Trust: Akeel Moosa; Dunedin Hospital: Annette Neylon, Hilda Mangos; East Kent Hospitals University NHS Foundation Trust: Jindriska Lindsay, Kamiran Saied, Christopher Pocock, Vijay Ratnayake; East Sussex Hospitals NHS Trust: Judy Beard, Satyajit Sahu; Epsom and St Helier University NHS Trust: Jane Mercieca; Falkirk and District Royal Infirmary: Christopher Brammer, Marie Hughes, Roderick Neilson; Glan Clwyd Hospital: Christine Hoyle, Earnest Heartin, Margaret Goodrick; Gloucestershire Royal Hospital: Adam Rye, Eve Blundell, Rebecca Frewin, Sally Chown; Great Western Hospital: Alex Sternberg, Atherton Gray, Norbert Blessing; Guys and St Thomas' Foundation Trust: Kavita Raj, Robert Carr; Hammersmith Hospital: Jiri Pavlu; Heatherwood and Wexham Park NHS foundation Trust: Mark Offer, Nicola Bienz, Nicola Philpott, Simon Moule; Hereford County Hospital: Lisa Robinson, Sara Willoughby; Herlev Hospital: Morten Krogh Jensen, Peter Moller, Ulrik Overgaard; Hillingdon Hospital: Riaz Janmohamed, Richard Kaczmarski; Hull Royal Infirmary: Christopher Carter, Sahra Ali; Ipswich Hospital NHS Trust: Andrew Hodson, Debo Ademokun; James Cook University Hospital: Angela Wood, Ray Dang; James Paget University Hospital: Cesar Gomez, Manzoor Mangi, Shala Sadullah; John Radcliffe Hospital: Paresh Vyas; Kettering General hospital: Isaac Wilson-Morkeh, Karyn Longmuir, Mark Kwan, Matthew Lyttelten; Leicester Royal Infirmary: Ann Hunter, Kaljit Bhuller, Murray Martin; Lincoln County Hospital: Kandeepan Saravanamuttu; Maidstone Hospital: Evangelia Dimitriadou, Richard Gale; Manchester Royal Infirmary: Eleni Tholouli, Guy Lucas, John Yin, Sarah Burns; Medway Maritime Hospital: Maadh Aldouri, Vivienne Andrews; Milton Keynes Hospital NHS Foundation Trust: Moez Dungarwalla; Monklands Hospital: Alaeddin Raafat, Charlotte Thomas, Jane Laird, John Murphy, Pamela Paterson; NHS Grampian: Dominic Culligan, Jane Tighe; New Cross Hospital: Abraham Jacob, Alan MacWhanell, Sunil Hada, Supratik Basu; Ninewells Hospital and Medical Center: Keith Gelly, Sudhir Tauro; Norfolk and Norwich University Hospital NHS Foundation Trust: Matthew Lawes; North Middlesex University Hospital: Neil Rabin; Northampton General Hospital: Angela Bowen, Sajan Mittal, Suchitra Krishnamurthy; Northwick Park Hospital: Nicki Panoskaltsis, Robert Ayto; Nottingham University Hospitals NHS Trust: Emma Dasgupta, Jenny Byrne, Nigel Russell, Simone Stokley; Odense University Hospital: Claus Marcher, Lone Friis, Poul Gram Hansen; Palmerston North Hospital: Bart Baker, Paul Harper; Peterborough District Hospital: Kanchan Rege, Muthuswamy Sivakumaran, Sateesh Nagumantry; Pinderfield General Hospital: David Wright, Kavita Patil, Mary Chapple, Paul Moreton; Poole General Hospital: Fergus Jack, Ram Jayaprakash; Princess Royal University Hospital: Bipin Vadhir; Queen Alexandra Hospital: Christopher Jones, Helen Dignum, Mary Ganczakowski, Robert Corser, Tanya Cranfield; Queen Elizabeth Hospital: Charles Craddock, Jim Murray; Queen Elizabeth Hospital Woolwich: Ana Duran; Queen Elizabeth Hospital, Norfolk: Jane Keidan, Lisa Cooke; Queens Hospital: Claire Hemmaway, Jane Stevens; Raigmore Hospital NHS Highland: Chris Lush, Joanne Craig, Peter Forsyth; Rigshospitalet: Carsten Niemann, Lars Kjeldsen, Ole Wei Bjerrum, Ove Juul Nielsen, Peter Kampmann; Rotherham General Hospital: Arun Alfred, Richard Went; Royal Berkshire Hospital: Henri Groch, Rebecca Sampson, Stuart Mucklow; Royal Bournemouth Hospital: Joseph Chacko, Rachel Hall; Royal Cornwall Hospital: Bryson Pottinger; Royal Devon and Exeter Hospital: Anthony Todd, Claudius Rudin, Loretta Ngu, Malcolm Hamilton; Royal Free Hospital: Archie Prentice, Panos Kottaridis; Royal Hallamshire Hospital: Chris Dalley, Harpreet Kaur, John Snowden, Sameer Tulpule; Royal Marsden Hospital: David Taussig, Mark Ethell; Royal Oldham: Allameddine Allameddine, David Osborne, Hayley Greenfield, Sumaya Elhanash, Vivek Sen; Royal Shrewsbury Hospital: Andreea Corcoz; Royal Surrey County Hospital: Elisabeth Grey-Davies, Johannes DeVos, Louise Hendry; Royal United Hospital: Charles Singer, Christopher Knechtli, Josephine Crowe; Russells Hall Hospital: Jeff Neilson, Savio Fernandes, Stephen Jenkins; Salford Royal Hospital: John Houghton, Rowena Thomas-Dewing, Simon Jowitt, Sonya Zaman; Salisbury Hospital NHS Foundation: Effie Grand, Jonathan Cullis, Louise Fraser, Tamara Everington; Sandwell Hospital: Farooq Wandoo, Yasmin Hasan; Singleton Hospital: Hamdi Sati, Saad Ismail; South Devon Healthcare NHS Foundation Trust: Deborah Turner, Nicholas Rymes, Patrick Roberts, Steve Smith; Southampton University Hospital NHS Trust: Deborah Richardson, Kim Orchard, Matthew Jenner; Southern General Hospital: Anne Morrison, Ian Macdonald; St Helens and Knowsley NHS Trust: Toby Nicholson; St James University Hospital: David Bowen; St Richards Hospital: Phillip Bevan, Sarah Janes; Stafford Hospital: Andrew Amos, Aurangzeb Razzak; Stoke Mandeville Hospital: Anne-Marie O'Hea, Jonathan Pattinson, Robin Aitchison; Sunderland Royal Hospital: Annette Nicolle, Scott Marshall, Yogesh Upadhye; Taunton and Somerset Foundation Trust: Sarah Allford, Simon Bolam; The Newcastle upon Tyne NHS Foundation Trust: Anne Lennard, Chris Williams, Gail Jones, Graham Jackson; University College London Hospitals: Andres Virchis, Asim Khwaja, Kwee Yong, Nishal Patel; University Hospital Aintree: Arpad Toth, Barbara Hammer, Barrie Woodcock, Lynny Yung, Walid Sadik; University Hospital Coventry and Warwickshire NHS Trust: Anand Lokare, Anton Borg, Beth Harrison, Mekkali Narayanan, Nicholas Jackson, Oliver Chapman, Sarah Nicolle, Shailesh Jobanputra, Syed Bokhari; University Hospital Lewisham: Naheed Mir; University Hospital of North Staffordshire NHS Trust: Deepak Chandra, Kamaraj Karunanithi, Neil Phillips, Richard Chasty, Srinivas Pillai; University Hospital of Wales: Caroline Alvares, Jonathan Kell, Steve Knapper; University Hospital of North Tees and Hartlepool: Philip Mounter, Philip Saunders, Zor Maung; University of Liverpool and Royal Liverpool University Hospital: Rahuman Salim, Richard Clark; Victoria Hospital NHS Fife: Kerri Davidson, Peter Williamson, Stephen Rogers; Waikato Hospital: Gillian Corbett, Humphrey Pullon, Shahid Islam; Wellington Hospital: Alwyn D'Souza, Huib Buyck, John Carter, Kentneth Romeril; Western General Hospital: PH Roddie, Peter Johnson; Wishaw General Hospital: Annielle Hung, Gila Helenglass; Worcestershire Royal Hospital: Elizabeth Maughan, Juliet Mills, Salim Shafeek; Worthing Hospital: Santosh Narat; York Hospital: Laura Munro, Lee Bond, Martin Howard; Ysbyty Gwynedd: David Edwards, James Seale.
